# Can Newts Cope with the Heat? Disparate Thermoregulatory Strategies of Two Sympatric Species in Water

**DOI:** 10.1371/journal.pone.0128155

**Published:** 2015-05-20

**Authors:** Monika Balogová, Lumír Gvoždík

**Affiliations:** 1 Institute of Biology and Ecology, Faculty of Science, P.J. Šafárik University, Košice, Slovakia; 2 Institute of Vertebrate Biology, Academy of Sciences of the Czech Republic, Brno, Czech Republic; University of Sao Paulo, BRAZIL

## Abstract

Many ectotherms effectively reduce their exposure to low or high environmental temperatures using behavioral thermoregulation. In terrestrial ectotherms, thermoregulatory strategies range from accurate thermoregulation to thermoconformity according to the costs and limits of thermoregulation, while in aquatic taxa the quantification of behavioral thermoregulation have received limited attention. We examined thermoregulation in two sympatric newt species, *Ichthyosaura alpestris* and *Lissotriton vulgaris*, exposed to elevated water temperatures under semi-natural conditions. According to a recent theory, we predicted that species for which elevated water temperatures pose a lower thermal quality habitat, would thermoregulate more effectively than species in thermally benign conditions. In the laboratory thermal gradient, *L*. *vulgaris* maintained higher body temperatures than *I*. *alpestris*. Semi-natural thermal conditions provided better thermal quality of habitat for *L*. *vulgaris* than for *I*. *alpestris*. Thermoregulatory indices indicated that *I*. *alpestris* actively thermoregulated its body temperature, whereas *L*. *vulgaris* remained passive to the thermal heterogeneity of aquatic environment. In the face of elevated water temperatures, sympatric newt species employed disparate thermoregulatory strategies according to the species-specific quality of the thermal habitat. Both strategies reduced newt exposure to suboptimal water temperatures with the same accuracy but with or without the costs of thermoregulation. The quantification of behavioral thermoregulation proves to be an important conceptual and methodological tool for thermal ecology studies not only in terrestrial but also in aquatic ectotherms.

## Introduction

Fast climate change poses a serious challenge to the current biota. The ability of organisms to withstand the impact of climate change primarily depends on their actual exposure to changed climatic conditions [1]. Many ectotherms are not passive to the variation of the thermal environment and reduce the exposure to environmental temperatures using behavioral thermoregulation [2]. Recent analysis confirmed the key role of this behavior in coping with climate change in terrestrial insects, amphibians, and squamate reptiles [3]. They also showed its limitation depending on the availability of cool refugia in ectotherm habitats, which suggests that lowland tropical and desert species are more vulnerable to a warming climate than temperate taxa [4–5]. However, the scarcity of information about the effectiveness of behavioral thermoregulation in aquatic organisms complicates understanding of the general trend in ectotherm exposure to climate change.

Although the physical characteristics of water, high heat capacity and fast thermal conductivity prevent small-bodied ectotherms from maintaining their *T*_b_ above or below temperatures of the surrounding medium, many aquatic organisms are capable of behavioral thermoregulation by moving across a thermally-stratified water column [6–7]. The ongoing rapid climate change increases the temperature in freshwater ecosystems providing, among other things, a new challenge for aquatic thermoregulators. Although the aquatic environment is more buffered against extreme temperatures than land, preferred body temperatures are generally lower in aquatic taxa than in their terrestrial counterparts [6, 8–10]. Accordingly, relatively small increases in temperature in shallow waters may markedly increase the proportion of available water temperatures above an ectotherm’s target thermoregulatory range. A recently published model [11] predicts an ecthotherm’s thermoregulation in a predominantly warm environment. The model assumes that the impact of suboptimally high body temperatures on organism performance is more severe than on the colder (ascending) part of the thermal performance curve [9]. If environmental temperatures frequently exceed an ectotherm’s target range, its thermoregulatory effectiveness should increase as the thermal quality of its habitat (proportion of operative temperatures outside the target range) decreases. Unfortunately, whether this prediction holds also for aquatic thermoregulators remains unknown.

In this study, we examined aquatic behavioral thermoregulation in two species of newts, *Ichthyosaura alpestris* and *Lissotriton vulgaris*, exposed to elevated environmental temperatures. Newts are suitable for this task for three reasons. First, adult newts use thermoregulatory behavior in water [12]. Second, thermal conditions in newt aquatic habitats, i.e. shallow pools and ponds, provide various opportunities for behavioral thermoregulation in the field [13]. Finally, although both species sometimes co-occur in water bodies, the Czech populations of *I*. *alpestris* largely occupy forested areas at altitudes above 400 m, while *L*. *vulgaris* commonly occurs in open landscapes well below and above this altitudinal level [14–16]. Hence, from a thermal ecology view, the variation in habitat use suggests diverse thermal requirements or thermoregulatory effectiveness and accordingly, the exposure to climate change in both newt species.

**Fig 1:**
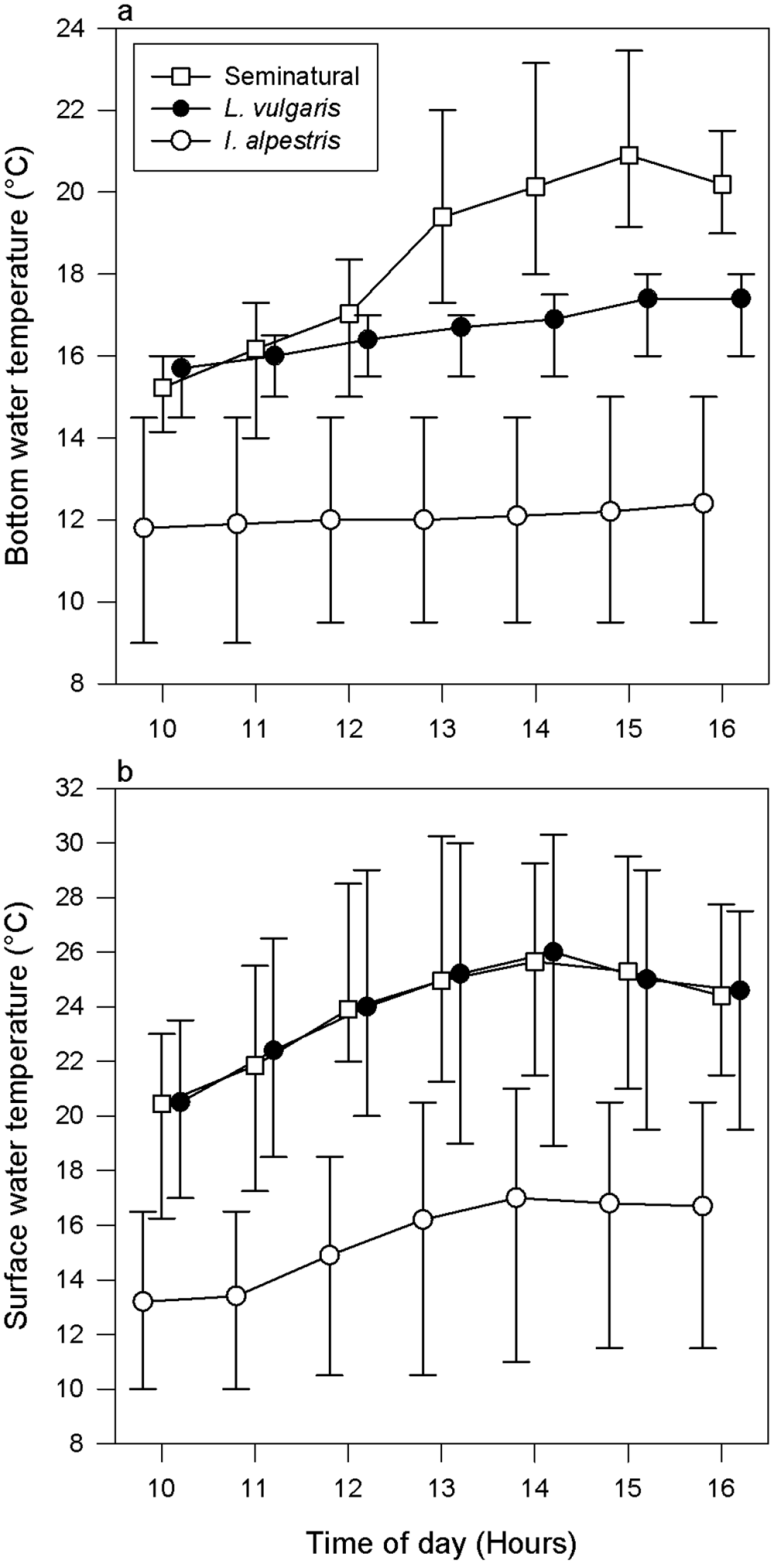
Water temperatures experienced by newts, *Ichthyosaura alpestris* and *Lissotriton vulgaris*, in native and semi-natural aquatic habitats between 6^th^ and 19^th^ June 2014. (a) Water temperatures at the maximum depth, (b) temperatures on water surface. Values (hourly means±extremes) are shifted horizontally to reduce overlap.

We predicted that elevated water temperatures would induce a higher thermoregulatory effort in newt species for which the thermal conditions pose a lower thermal quality environment than for the species with thermal requirements largely matching available water temperatures. To test this prediction, we subjected both species to the same thermal conditions, which were slightly warmer for *L*. *vulgaris* and substantially warmer for *I*. *alpestris* than water temperatures in their native habitats (Fig 1). Local climate warming trend [17] indicate that populations of the later species would be exposed to these temperatures in the near future.

## Materials and Methods

### Study Species and Maintenance

*Lissotriton vulgaris* and *I*. *alpestris* are small to medium-sized newts (total length up to 90 and 120 mm, respectively), which are widely distributed across Europe [14–15]. Both species share a similar lifestyle. Newts usually spend part of the year in water (April–June) and the rest of the year on land. During their aquatic phase, they occupy a variety of still waters, such as pools, ponds, and lakes. Although newts are largely crepuscular and nocturnal, they are also active during daytime. Their aquatic diet consists of diverse invertebrates, ranging from planktonic crustaceans to oligochaetes and chironomid larvae. Preferred body temperatures provide the best compromise for various thermal reaction norms and performance curves of fitness-related traits in newts [18–19].

We captured adult newts (*I*. *alpestris*: *n* = 30 [1:1 sex ratio], snout-vent length [SVL] = 45.7± 3.7 mm [mean±SD]; *L*. *vulgaris*: *n* = 30 [1:1 sex ratio], SVL = 38.9± 2.8 mm) from two water bodies, located 4 km apart, near Jihlava, Czech Republic. The pool occupied by *I*. *alpestris* was located in a forest at 600 m altitude (49°23'18"N, 15°30'48"E), whereas the pool with *L*. *vulgaris* was situated in open habitat at an altitude of 560 m (49°22'56"N, 15°33'10"E). Both populations were chosen according to their high abundance to provide sufficient sample size and a minimum distance from each other.

Newts (one male and one female) were placed in plastic aquaria filled with 18 l of non-chlorinated well water. Each aquarium was equipped with aquatic weeds (*Egeria densa*) and a piece of Styrofoam allowing newts to leave water. Aquaria were placed in a room with a natural light:dark cycle and diel thermal fluctuations 12–22°C. Temperatures were selected according to the most frequent temperatures in their natural habitat [17]. Newts were fed with live *Chironomus* larvae, *Tubifex* worms, and *Eisenia* eartworms once or twice per week. Water (50% of total volume) was changed on weekly basis.

### Preferred Body Temperatures

Preferred body temperatures, i.e., body temperatures maintained by an ectotherm under the absence of thermoregulatory constraints, were measured in a stainless steel tank (240×60×60 cm high). The tank was divided into three longitudinal lanes. Each lane consists of 12 partially separated compartments (20×20 cm) with different water temperatures. The bottom of each compartment was equipped with Peltier modules that were connected together with a heat recuperation system to a programmable control unit, which allowed for the creation of a horizontal thermal gradient in water. We used thermal gradients between 8 and 30°C in 2±0.5°C steps. The tank was filled with non-chlorinated well water up to 3 cm. Mild aeration promoted thermal homogenization of water in each compartment. The tank was placed in a room at 18±0.5°C. Series of fluorescent daylight tubes above the tanks provided light intensity of 300 lx on the water surface.

Since reproduction and satiation level influence *T*_p_ in newts [20–21], we used non-reproductive and fully fed individuals for its measurements. In the field, most newts contain food in their stomachs [22], and so we considered the *T*_p_ of fully fed individuals more ecologically realistic than the *T*_p_ of hungry newts. Three randomly chosen newts were placed individually into the tank lane 15 hrs before the beginning of trials. Their behavior was recorded using a digital camera (12 fps; MTV-63S80H-A-ICR-R, Mintron, Taipei, Taiwan) connected to a PC surveillance system (V-Guard RT4, Chateau, Taipei, Taiwan) between 10:00 and 17:00. Water was changed after each trial. Six thermistor probes connected to dataloggers (resolution 0.1°C; HOBO, Onset Computer, Bourne, MA, USA) recorded the minimum and maximum temperatures in each lane to monitor the stability of the thermal gradient during trials.

Later, video recordings were checked and newt horizontal positions in the thermal gradient (compartment) were recorded at 10 min intervals. Since the *T*_b_ of small ectotherms quickly equilibrate with the temperature of the surrounding medium [23], we estimated newt *T*_b_ indirectly from the known water temperatures in a given compartment. Data from newts that were inactive or left the water for a prolonged amount of time (>90%) were discarded from further analyses. From individual *T*_b_ distribution (*n* = 216), we calculated the lower and upper *T*_p_ boundaries as the 10th and 90th percentiles, respectively. We assumed that these values delimit the target range of *T*_b_ that newts aim to achieve by behavioral thermoregulation.

### Operative and Body Temperatures

To compare behavioral thermoregulation of both species under elevated temperatures and without the confounding influence of biotic factors, we performed thermoregulatory trials in tanks under semi-natural conditions. We used 14 fiberglass tanks (90×63×47 cm high) that were half-buried in soil in two rows in an east-west direction. The bottom of each tank was profiled (15 cm flat, 60 cm inclined, 15 cm flat) allowing bottom-walking newts to move across the water columns. Tanks were filled with non-chlorinated well water up to 45 cm. Each 5 cm of water column was marked on the bottom to record the newt’s vertical position. Two marginal and one middle tank contained series of nine waterproofed dataloggers (resolution 0.5°C; DS1921G-F5, Maxim Integrated Products, Sunnyvale, CA, USA) that recorded water temperatures at each 5 cm of the water column at 30 min intervals. The difference between cages was always ≤1°C, which indicates that the dataloggers sufficiently mapped thermal conditions in the experimental containers. Since the *T*_b_ of small ectotherms quickly equilibrate with water temperatures, the physical properties of newt bodies have a minor influence on their equilibrium *T*_b_. Accordingly, we considered water temperatures as the reliable *T*_e_ estimates, i.e., equilibrium temperatures of an inanimate object with the same physical properties as the studied ectotherm [24], in both species. The vertically stratified *T*_e_ profile represents a “null-distribution” of *T*_b_s that non-thermoregulating newts would achieve.

To evaluate newt thermoregulatory behavior, we placed randomly chosen newts individually into experimental tanks two hours before the beginning of each trial. One person (MB) walked slowly along tanks and recorded newt vertical positions (resolution 5 cm) at hourly intervals between 10:00 and 17:00. Our previous observations confirmed that the presence of an observer had no influence on newt behavior. Similarly to *T*_p_, we estimated newt *T*_b_ indirectly from their vertical position on the profiled bottom with known water temperature. All experiments were performed between 3^rd^ and 19^th^ June 2014.

### Thermoregulatory Indices

Using information from the *T*_p_ range, *T*_e_ and *T*_b_, three thermoregulatory indices were calculated [24, 25]. 1. Thermal quality of habitat: The deviation of mean *T*_e_ from the *T*_p_ range. If *T*_e_ falls within the range, *d*_e_ equals zero. The low index value indicates high thermal quality of habitat for focal species and vice versa. Since *T*_e_ varied substantially in time, the index was calculated for each individual at hourly intervals. 2. Accuracy of thermoregulation (*d*_b_): Calculated in the same way as *d*_e_ with *T*_b_ instead of *T*_e_. 3. Effectiveness of thermoregulation (*E*): The index was originally calculated as *E* = *d*_b_/*d*_e_. Since the ratio may provide poor quantification of thermoregulation, we used an alternative calculation as *E* = *d*_e_–*d*_b_ [25]. Values around zero indicate thermoconformity, positive values active thermoregulation, whereas negative values suggest the active avoidance of target temperatures.

### Statistical Analyses

Prior to model testing, we performed exploratory analyses involving the detection of possible outliers, non-normality, heterogeneity, non-independence, and the covariation of explanatory variables. Since we obtained repeated measurements of *T*_e_ and *T*_b_ during trials, their variation was analyzed using a general linear mixed model for short time series [26]. The full model consists of three fixed factors and their interactions, species, time of day, and date of measurement. The model variance structure was improved using date of measurement (*T*_e_ analysis) or species (*T*_b_ analysis) as the variance covariate. Individual identity (intercept) and individual identity in time (intercept and slope) were added as random factors. The optimal model structure was selected using the likelihood approach, i.e. we compared the model maximum likelihood for fixed factors and the restricted maximum likelihood for random factors. Since *T*_p_ boundaries and thermoregulatory indices did not meet some of the assumptions (outliers, non-normality) mentioned above, we applied a robust randomization (permutation) approach for statistical inference. The full model contained the same factors as above. The optimal model structure was selected according to factor variance components, i.e. factors that explained no variance in the examined trait was dropped from the model. In all randomization tests, we used 9999 permutations to calculate *P* values. Confidence intervals for sample means were obtained using a non-parametric bootstrap procedure (9999 replications). Statistical analyses were performed using ‘perm’ [27], ‘nlme’ [28] and ‘boot’ [29] libraries in R [30] and ‘PERMANOVA’ package for PRIMER (version 6; Primer E, Lutton, UK).

## Results

**Fig 2:**
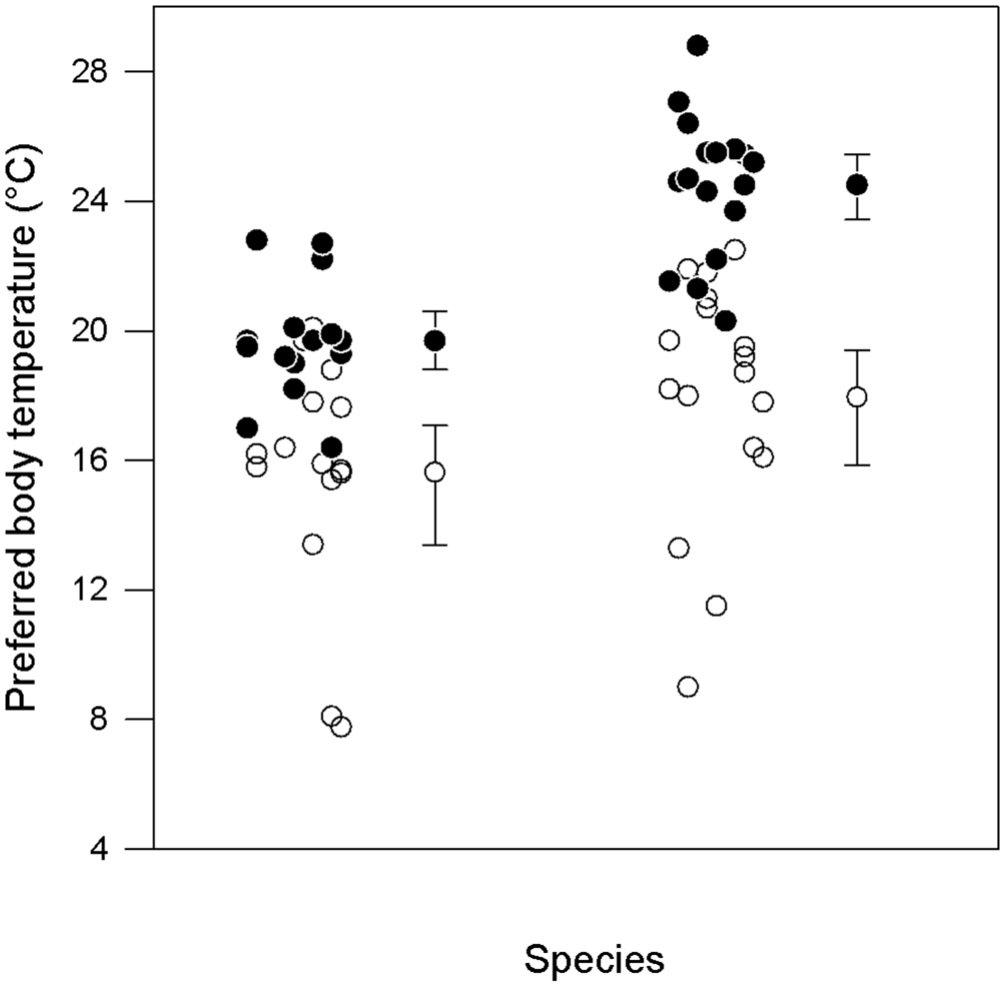
Lower and upper boundaries (mean ± 95%CI) of preferred body temperatures in two newt species, *Ichthyosaura alpestris* and *Lissotriton vulgaris*. Boundaries were calculated from 10^th^ and 90^th^ percentiles of individual distributions. Datapoints are jittered horizontally to reduce overlap.

Preferred body temperatures were measured in 15 individuals of each species. Three *I*. *alpestris* and one *L*. *vulgaris* remained motionless during the whole trial, and thus their data were removed from further analyses. Since factors ‘sex’ and ‘sex×species’ explained only minor variations in the *T*_p_ characteristic (mean, lower boundary, and upper boundary), they were dropped from the minimum adequate model. *Ichthyosaura alpestris* maintained a lower mean *T*_p_ and upper *T*_p_ boundary than *L*. *vulgaris* (mean: *I*. *alpestris*: 17.9°C, 95% CI [16.7, 18.7]; *L*. *vulgaris*: 21.7°C, 95% CI [20.5, 22.5]; pseudo-*F*_1,30_ = 12.42, *P* < 0.001; upper boundary: pseudo-*F*_1,30_ = 17.28, *P* < 0.001; Fig 2). Between-species variation in lower *T*_p_ boundary was statistically non-significant (pseudo-*F*_1,30_ = 3.49, *P =* 0.07; Fig 2).

**Fig 3:**
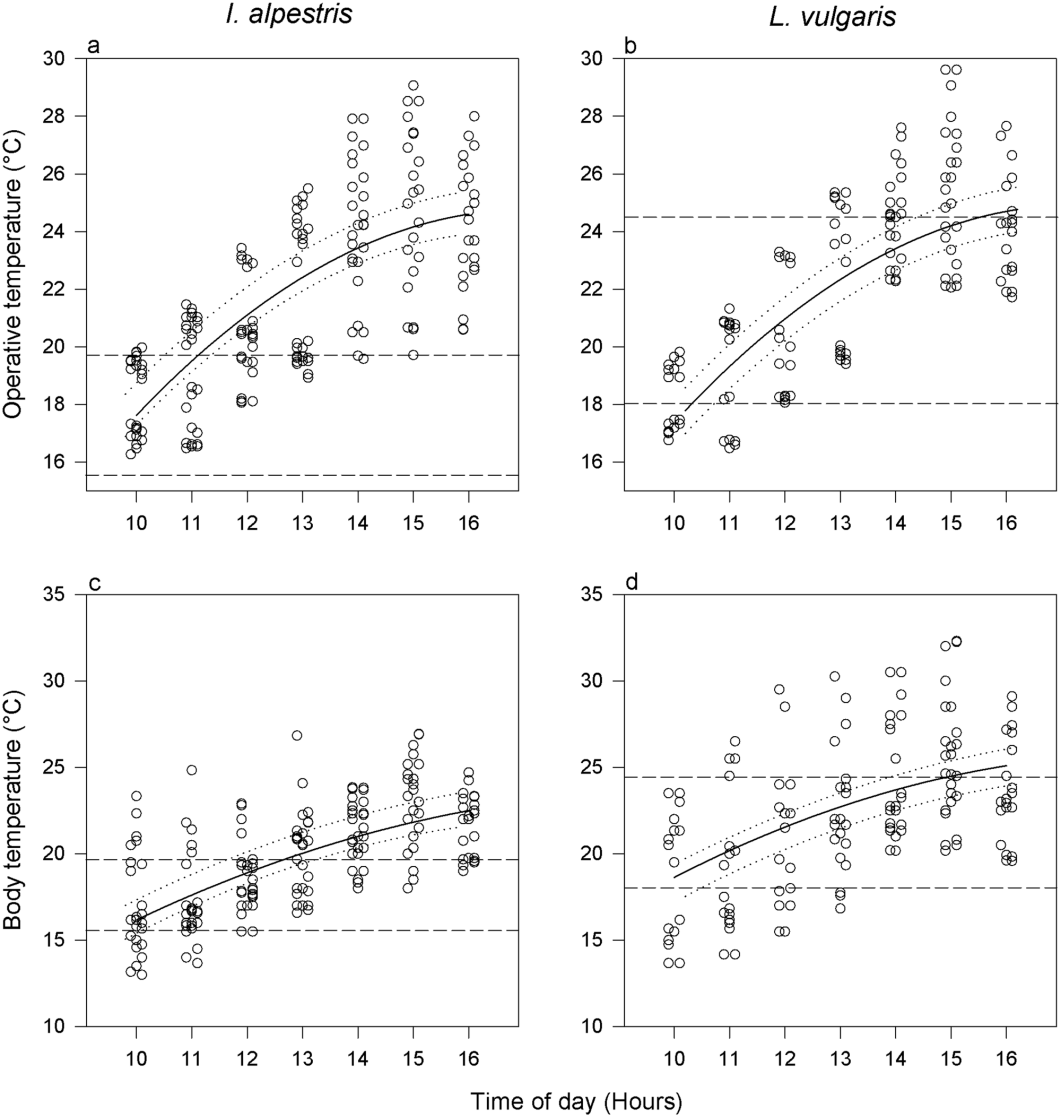
Operative and body temperatures as a function of the time of day in adult *Ichthyosaura alpestris* and *Lissotriton vulgaris* in semi-natural settings during June. Datapoints (mean operative temperatures across the water columns and individual body temperatures) are jittered horizontally to reduce overlap. Data are fitted with a general linear mixed model (±95% CI). See Table 2 for model parameters. Horizontal dashed lines denote the lower and upper *T*_p_ boundaries.

Operative temperatures increased during daytime in a curvilinear fashion (Fig 3a and 3b; Table 1). Both species experienced a similar *T*_e_ (χ^2^ = 0.10, *P =* 0.75). The daily course of *T*_b_ showed the same trend as *T*_e_ (Fig 3c and 3d; Table 1). Mean *T*_b_ were consistently lower in *I*. *alpestris* than in *L*. *vulgaris* (χ^2^ = 13.13, *P <* 0.001; Table 2).

**Table 1:** Fixed factor parameters of the minimum adequate models explaining the effect of daytime and species on operative and body temperatures of adult newts, *Ichthyosaura alpestris* and *Lissotriton vulgaris*, during June.

Trait	Factor	Value±SE	df	*t*	*P*
Operative temperatures	Intercept	-17.076±3.044	286	-5.61	<0.001
	Linear effect of time	4.912±0.472	286	10.40	<0.001
	Quadratic effect of time	-0.144±0.018	286	-7.96	<0.001
	Species	0.163±0.519	54	0.31	0.75
Body temperatures	Intercept	-9.870±4.858	286	-2.03	0.043
	Linear effect of time	3.538±0.757	286	4.67	<0.001
	Quadratic effect of time	-0.095±0.029	286	-3.26	0.001
	Species	2.591±0.688	54	3.76	<0.001

See Fig 3 for a graphical representation of results.

**Table 2:** Summary statistics of thermoregulatory characteristics of adult newts, *Ichthyosaura alpestris* and *Lissotriton vulgaris*.

Trait	Species			
	*I*. *alpestris*		*L*. *vulgaris*	
	*n =* 30		*n =* 26	
	Mean	95% CI	Mean	95% CI
Operative temperature (°C)	21.8	21.1–22.5	21.9	21.3–22.6
Body temperature (°C)	19.8	19.1–20.5	22.8	21.5–24.3
Thermal quality of habitat (°C)	2.8	2.2–3.4	0.9	0.7–1.2
Accuracy of thermoregulation (°C)	1.5	1.1–2.0	1.4	1.0–2.2
Effectiveness of thermoregulation (°C)	1.3	0.9–1.7	-0.4	-1.0–0

See text for calculations of thermoregulatory indices.

Given the variation in *d*_e_, semi-natural conditions provided lower thermal quality for *I*. *alpestris* than for *L*. *vulgaris* (pseudo-*F*_1,64_ = 24.22, *P <* 0.001; Table 2). Newts maintained their *T*_b_ within the *T*_p_ range with the same accuracy in both species (pseudo-*F*_1,61_ = 0.01, *P =* 0.92). Accordingly, the *E* index markedly differed between species (pseudo-*F*_1, 64_ = 31.17, *P <* 0.001). The index values indicate active thermoregulation in *I*. *alpestris* and thermoconformity in *L*. *vulgaris* (Table 2). Thermoregulatory effectiveness was consistent during the day in both species (Hour: pseudo-*F*_6,267_ = 1.29, *P =* 0.27; Hour × Species: pseudo-*F*_6,267_ = 1.51, *P =* 0.17). Newt individual identity explained 23% of total variance in *E* (pseudo-*F*_54,267_ = 2.81, *P <* 0.001).

## Discussion

Unlike the long research tradition in terrestrial ectotherms, the quantification of behavioral thermoregulation in aquatic taxa has received surprisingly little attention. We demonstrated that two sympatric newt species adopted disparate thermoregulatory strategies, active thermoregulation and thermoconformity, to cope with elevated temperatures in water. The species exposed to the lower quality thermal habitat thermoregulated with a higher effort than species in thermally more benign conditions, which corroborates the prediction of the current theory [11]. Thermoconforming *L*. *vulgaris* maintained a higher *T*_p_ and within a wider range than the thermoregulating *I*. *alpestris*, which suggests that the variation in thermoregulatory strategies largely reflects the variation in their *T*_p_ ranges. Both strategies allowed newts to keep their *T*_b_ within the target range with the same accuracy but with or without the costs of thermoregulation.

Although the costs of thermoregulation were not quantified in this study, the available information provides at least a qualitative view on this issue. Our results showed that the behavioral thermoregulation forced *I*. *alpestris* to cooler and deeper waters than *L*. *vulgaris*. Accordingly, the reduced activity space may restrict the opportunity to find food and increase the time and energy costs of aerial breathing because of prolonged traveling distances to the water surface [18]. Contrary to thermoregulatory costs in terrestrial taxa, which include time and energy spent thermoregulating, predation risk, or missed opportunity [31], the presumed costs in thermoregulating newts are indirect, i.e., restricted foraging space and the increased time and energy demands of aerial breathing. Further research will show how the indirect costs of thermoregulation affect the ultimate currency, fitness or its components, in newts.

Why did sympatric species achieve the same accuracy of thermoregulation by adopting disparate thermoregulatory strategies? As mentioned above, these strategies largely reflect between species variation in *T*_p_ ranges. The exact source of *T*_p_ variation is unknown. Among species, *T*_p_ may be affected by their independent phylogenetic history, though the evolutionary rates (the change in an evolutionary lineage over time) of *T*_p_ are rather low in newts [32]. Within-species, thermally-induced developmental plasticity [33] may also contribute to this result. This suggests that newt thermoregulatory strategies in an aquatic environment is primarily determined by factors limiting the variation in target temperatures, which newts aim to achieve.

The *E* values indicate that under elevated water temperatures *I*. *alpestris* was able to maintain *T*_b_ that were on average 1.3°C below the mean *T*_e_. In comparison with *E* values in terrestrial ectotherms [34], the value is fairly low suggesting that newts thermoregulated with much lower effectiveness in water. Unfortunately, we are not aware of any study reporting estimates of thermoregulatory effectiveness in other aquatic taxa. Hence, further research will show whether newt thermoregulation is less effective than in terrestrial taxa because of the physical characteristics of water, phylogenetic distance, or other factors.

While both species effectively coped with elevated water temperatures, further warming would affect thermoregulating *I*. *alpestris* more than thermoconforming *L*. *vulgaris*, not only because of the costs of thermoregulation, but also of prolonged time, when thermal conditions prevent behavioral thermoregulation. The negative effect of thermal constraints could be avoided in three ways. First, newts could simply prefer deeper water bodies, which provide sufficiently cold refugees. However, this depends on the availability of suitable water bodies, which should be not only deep enough, but provide other suitable biotic and abiotic conditions [16]. Second, newts could leave the aquatic habitat before it became too warm. However, newts from southern populations respond to hot and dry weather by staying in water year-round rather than by reducing their aquatic period [35]. Finally, newts could shift their *T*_p_ range. Given their long generation time and evolutionary rates of *T*_p_, the only viable alternative is a plastic response (see above). However, whether the plastic shift induced during embryonic development [33] persists for the rest of life is unknown. In addition, the magnitude of a seasonal plastic response is relatively low [13] and information about its costs and limits [36] are lacking. Taking into account also the negative influence of elevated water temperatures on larvae [37], *I*. *alpestris* would likely respond to climate warming by restricting its occurrence to cooler habitats [3, 5].

Our study was performed in seminatural settings, which eliminated many unwanted factors at the expense of lower ecological realism. Alpine newts were intentionally exposed to much higher water temperatures than in their native habitat, and thus it can be argued that our results were affected by ecologically unrealistic thermal conditions. However, the maximum water temperatures were not higher than temperatures that newts experienced in the sun-exposed pool located close to the population studied [17]. Hence, we considered the thermal profile used a reasonable estimate of conditions that newts would face as a result of deforestation, heat waves [38], or local climate change in the near future.

## Conclusions

Our study provides a rare attempt to quantify thermoregulatory strategies in aquatic ectotherms. Adopting the same approach as terrestrial ecologists [24], our results revealed two attributes that aquatic temperate newts share surprisingly not with temperate, but with tropical terrestrial ectotherms. First, while temperate ectotherms thermoregulate effectively despite thermally challenging conditions [25, 39–40], tropical species frequently become thermoconformers in thermoregulatory costly habitats [24, 41]. Hence, the variation in thermoregulatory strategies of aquatic newts is more similar to tropical rather than temperate species on land. Second, the major task for temperate thermoregulators is to heat the body above the average environmental temperature, whereas tropical thermoregulators have to solve the opposite issue, i.e. how to cool their *T*_b_ [2, 11]. We showed that under elevated water temperatures, one newt species was faced with the same task as tropical ectotherms. Hence, our study challenged the tropical-temperate patterns in behavioral thermoregulation. This proves that the quantification of thermoregulatory strategies in at least some aquatic ectotherms provides a similarly important tool for understanding their thermal ecology and responses to climate change as in terrestrial taxa.

## References

[1] Williams SE, Shoo LP, Isaac JL, Hoffmann AA, Langham G. Towards an integrated framework for assessing the vulnerability of species to climate change. PLoS Biol. 2008; 6: 2621–2626. 10.1371/journal.pbio.0060325 19108608 PMC2605927

[2] Kearney M, Shine R, Porter WP. The potential for behavioral thermoregulation to buffer "cold-blooded" animals against climate warming. Proc Natl Acad Sci USA. 2009; 106: 3835–3840. 10.1073/pnas.0808913106 19234117 PMC2656166

[3] Sunday JM, Bates AE, Kearney MR, Colwell RK, Dulvy NK, Longino JT, et al Thermal-safety margins and the necessity of thermoregulatory behavior across latitude and elevation. Proc Natl Acad Sci USA. 2014; 111: 5610–5615. 10.1073/pnas.1316145111 24616528 PMC3992687

[4] Huey RB, Deutsch CA, Tewksbury JJ, Vitt LJ, Hertz PE, Perez HJ, et al Why tropical forest lizards are vulnerable to climate warming. Proc R Soc B. 2009; 276: 1939–1948. 10.1098/rspb.2008.1957 19324762 PMC2677251

[5] Sinervo B, Mendez-de-la-Cruz F, Miles DB, Heulin B, Bastiaans E, Villagran-Santa Cruz M, et al Erosion of lizard diversity by climate change and altered thermal niches. Science. 2010; 328: 894–899. 10.1126/science.1184695 20466932

[6] Hutchison VH, Dupré RK. Thermoregulation In: Feder ME, Burggren WW, editors. Environmental physiology of amphibians. Chicago: University of Chicago Press; 1992 pp. 206–249.

[7] Lancaster J, Downes BJ. Aquatic entomology. Oxford: Oxford University Press; 2013.

[8] Johnson JA, Kelsch SW. Effects of evolutionary thermal environment on temperature-preference relationships in fishes. Environ Biol Fishes. 1998; 53: 447–458.

[9] Martin TL, Huey RB. Why "suboptimal" is optimal: Jensen's inequality and ectotherm thermal preferences. Am Nat. 2008; 171: E102–E118. 10.1086/527502 18271721

[10] Azócar DLM, Vanhooydonck B, Bonino MF, Perotti MG, Abdala CS, Schulte JA, et al Chasing the Patagonian sun: comparative thermal biology of *Liolaemus* lizards. Oecologia. 2013; 171: 773–788. 10.1007/s00442-012-2447-0 23011849

[11] Vickers M, Manicom C, Schwarzkopf L. Extending the cost-benefit model of thermoregulation: high-temperature environments. Am Nat. 2011; 177: 452–461. 10.1086/658150 21460567

[12] Marek V, Gvoždík L. The insensitivity of thermal preferences to various thermal gradient profiles in newts. J Ethol. 2012; 30: 35–41.

[13] Hadamová M, Gvoždík L. Seasonal acclimation of preferred body temperatures improves the opportunity for thermoregulation in newts. Physiol Biochem Zool. 2011; 84: 166–174. 10.1086/658202 21460527

[14] Roček Z, Joly P, Grossenbacher K. *Triturus alpestris* (Laurenti, 1768)—Bergmolch In: Grossenbacher K, Thiesmeier B, editors. Handbuch der Amphibien und Reptilien Europas. Schwanzlurche II A. Wiesbaden: Aula Verlag; 2003 pp. 607–656.

[15] Schmidtler JF, Franzen M. *Triturus vulgaris* (Linnaeus, 1758)—Teichmolch In: Grossenbacher K, Thiesmeier B, editors. Handbuch der Amphibien und Reptilien Europas. Schwanzlurche II B. Wiesbaden: Aula Verlag; 2004 pp. 847–967.

[16] Van Buskirk J. Local and landscape influence on amphibian occurrence and abundance. Ecology. 2005; 86: 1936–1947.

[17] Dvořák J, Gvoždík L. Adaptive accuracy of temperature oviposition preferences in newts. Evol Ecol. 2010; 24: 1115–1127.

[18] Šamajová P, Gvoždík L. The influence of temperature on diving behaviour in the alpine newt, *Triturus alpestris* . J Therm Biol. 2009; 34: 401–405.

[19] Kurdíková V, Smolinský R, Gvoždík L. Mothers matter too: Benefits of temperature oviposition preferences in newts. PLoS ONE. 2011; 6: e23842 10.1371/journal.pone.0023842 21887330 PMC3161085

[20] Gvoždík L. Postprandial thermophily in the Danube crested newt, *Triturus dobrogicus* . J Therm Biol. 2003; 28: 545–550.

[21] Gvoždík L. Does reproduction influence temperature preferences in newts? Can J Zool. 2005; 83: 1038–1044.

[22] Griffiths RA, Mylotte VJ. Microhabitat selection and feeding relations of smooth and warty newts, *Triturus vulgaris* and *T*. *cristatus*, at an upland pond in mid-Wales. Holarct Ecol. 1987; 10: 1–7.

[23] Lutterschmidt WI, Hutchison VH. The critical thermal maximum: data to support the onset of spasms as the definitive end point. Can J Zool. 1997; 75: 1553–1560.

[24] Hertz PE, Huey RB, Stevenson RD. Evaluating temperature regulation by field-active ectotherms: the fallacy of the inappropriate question. Am Nat. 1993; 142: 796–818. 10.1086/285573 19425957

[25] Blouin-Demers G, Weatherhead PJ. Thermal ecology of black rat snakes (*Elaphe obsoleta*) in a thermally challenging environment. Ecology. 2001; 82: 3025–3043.

[26] Zuur A, Ieno EN, Walker N, Saveliev AA, Smith GM. Mixed effects models and extensions in ecology with R. New York: Springer; 2009.

[27] Fay MP, Shaw PA. Exact and asymptotic weighted logrank tests for interval censored data: the interval R package. J Stat Soft. 2010; 36: 1–34. 10.18637/jss.v036.i02 PMC4184046 25285054

[28] Pinheiro J, Bates D, DebRoy S, Sarkar D, R Core Team. nlme: linear and nonlinear mixed effects models; 2014 Available: http://CRAN.R-project.org/package=nlme.

[29] Canty A, Ripley B. boot: Bootstrap R (S-Plus) Functions; 2014. Available: http://CRAN.R-project.org/package=boot.

[30] R Development Core Team. R: a language and environment for statistical computing, Version 3.1.1; 2014. Available: http://www.R-project.org.

[31] Angilleta MJ. Thermal adaptation. Oxford: Oxford University Press; 2009.

[32] Gvoždík L. Mismatch between ectotherm thermal preferenda and optima for swimming: a test of the evolutionary pace hypothesis. Evol Biol. 2015; 42: 137–145.

[33] Smolinský R, Gvoždík L. Interactive influence of biotic and abiotic cues on the plasticity of preferred body temperatures in a predator-prey system. Oecologia. 2012; 170: 47–55. 10.1007/s00442-012-2283-2 22358997

[34] Blouin-Demers G, Nadeau P. The cost-benefit model of thermoregulation does not predict lizard thermoregulatory behavior. Ecology. 2005; 86: 560–566.

[35] Fasola M, Canova L. Residence in water by the newts *Triturus vulgaris*, *T*. *cristatus* and *T*. *alpestris* in a pond in northern Italy. Amphib Reptil. 1992; 13: 227–233.

[36] Auld JR, Agrawal AA, Relyea RA. Re-evaluating the costs and limits of adaptive phenotypic plasticity. Proc R Soc B. 2010; 277: 503–511. 10.1098/rspb.2009.1355 19846457 PMC2842679

[37] Smolinský R, Gvoždík L. Does developmental acclimatization reduce the susceptibility to predation in newt larvae? Biol J Linn Soc. 2013; 108: 109–115.

[38] Smolinský R, Gvoždík L. Effect of temperature extremes on the spatial dynamics of predator—prey interactions: A case study with dragonfly nymphs and newt larvae. J Therm Biol. 2014: 39: 12–16.

[39] Gvoždík L. To heat or to save time? Thermoregulation in the lizard, *Zootoca vivipara* (Squamata: Lacertidae) in different thermal environments along an altitudinal gradient. Can J Zool. 2002; 80: 479–492.

[40] Valdecantos S, Martinez V, Lobo F, Cruz FB. Thermal biology of *Liolaemus* lizards from the high Andes: Being efficient despite adversity. J Therm Biol. 2013; 38: 126–134.

[41] Shine R, Madsen T. Is thermoregulation unimportant for most reptiles? An example using water pythons (*Liasis fuscus*) in tropical Australia. Physiol Zool. 1996; 69: 252–269.

