# Correction: Can Newts Cope with the Heat? Disparate Thermoregulatory Strategies of Two Sympatric Species in Water

**DOI:** 10.1371/journal.pone.0130918

**Published:** 2015-06-12

**Authors:** Monika Balogová, Lumír Gvoždík

Fig 2 is incorrect. The species category labels on the horizontal axis are inadvertently missing. The authors have provided a corrected version here.

**Fig 2:**
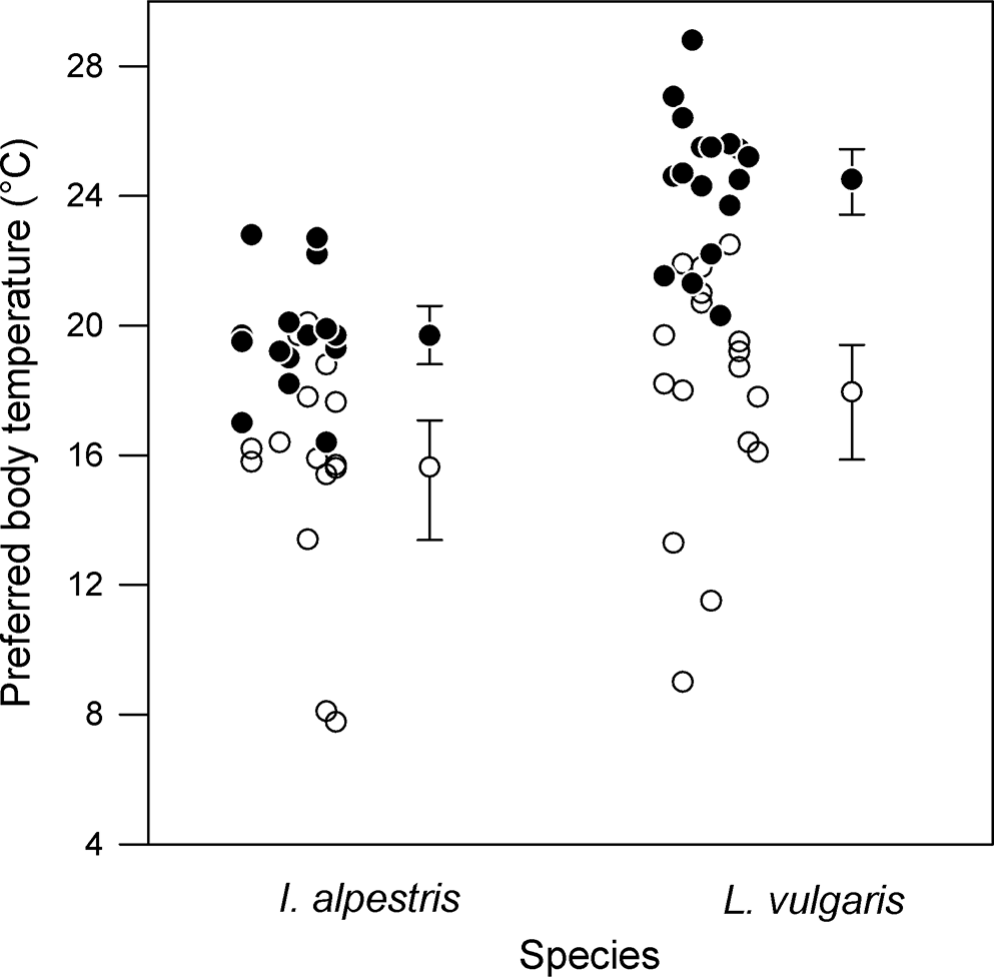
Lower and upper boundaries (mean ± 95%CI) of preferred body temperatures in two newt species, *Ichthyosaura alpestris* and *Lissotriton vulgaris*. Boundaries were calculated from 10^th^ and 90^th^ percentiles of individual distributions. Datapoints are jittered horizontally to reduce overlap.
